# Expression profiling of cancerous and normal breast tissues identifies microRNAs that are differentially expressed in serum from patients with (metastatic) breast cancer and healthy volunteers

**DOI:** 10.1186/bcr3127

**Published:** 2012-02-21

**Authors:** Eleni van Schooneveld, Maartje CA Wouters, Ilse Van der Auwera, Dieter J Peeters, Hans Wildiers, Peter A Van Dam, Ignace Vergote, Peter B Vermeulen, Luc Y Dirix, Steven J Van Laere

**Affiliations:** 1 Department of Oncology, University Hospitals Leuven and Catholic University Leuven, Herestraat 49, Leuven, B3000 Belgium; 2 Translational Cancer Research Unit, GZA Hospitals St-Augustinus, Oosterveldlaan 24, Antwerp, B2610 Belgium; 3 Radboud University Nijmegen Medical Center, Postbus 9101, Nijmegen, 6500 HB, The Netherlands; 4 Department of Medical Oncology, University Hospital Antwerp, Wilrijkstraat 10, B2650, Antwerp, Belgium

## Abstract

**Introduction:**

MicroRNAs (miRNAs) are a group of small noncoding RNAs involved in the regulation of gene expression. As such, they regulate a large number of cellular pathways, and deregulation or altered expression of miRNAs is associated with tumorigenesis. In the current study, we evaluated the feasibility and clinical utility of circulating miRNAs as biomarkers for the detection and staging of breast cancer.

**Methods:**

miRNAs were extracted from a set of 84 tissue samples from patients with breast cancer and eight normal tissue samples obtained after breast-reductive surgery. After reverse transcription and preamplification, 768 miRNAs were profiled by using the TaqMan low-density arrays. After data normalization, unsupervised hierarchical cluster analysis (UHCA) was used to investigate global differences in miRNA expression between cancerous and normal samples. With fold-change analysis, the most discriminating miRNAs between both tissue types were selected, and their expression was analyzed on serum samples from 20 healthy volunteers and 75 patients with breast cancer, including 16 patients with untreated metastatic breast cancer. miRNAs were extracted from 200 μl of serum, reverse transcribed, and analyzed in duplicate by using polymerase chain reaction (qRT-PCR).

**Results:**

UHCA showed major differences in miRNA expression between tissue samples from patients with breast cancer and tissue samples from breast-reductive surgery (*P *< 0.0001). Generally, miRNA expression in cancerous samples tends to be repressed when compared with miRNA expression in healthy controls (*P *= 0.0685). The four most discriminating miRNAs by fold-change (miR-215, miR-299-5p, miR-411, and miR-452) were selected for further analysis on serum samples. All miRNAs at least tended to be differentially expressed between serum samples from patients with cancer and serum samples from healthy controls (miR-215, *P *= 0.094; miR-299-5P, *P *= 0.019; miR-411, *P *= 0.002; and miR-452, *P *= 0.092). For all these miRNAs, except for miR-452, the greatest difference in expression was observed between serum samples from healthy volunteers and serum samples from untreated patients with metastatic breast cancer.

**Conclusions:**

Our study provides a basis for the establishment of miRNAs as biomarkers for the detection and eventually staging of breast cancer through blood-borne testing. We identified and tested a set of putative biomarkers of breast cancer and demonstrated that altered levels of these miRNAs in serum from patients with breast cancer are particularly associated with the presence of metastatic disease.

## Introduction

MicroRNAs (miRNAs) are a group of small (20 to 25 nt) noncoding RNAs able to regulate gene expression posttranscriptionally by binding to the 3'-untranslated region (UTR) of target mRNAs [1-3]. Since the initial discovery in *Caenorhabditis elegans*, more than 1,000 human miRNAs have been described, each of them targeting about 100 different mRNA molecules [4-6]. In this way, approximately 30% of all human genes are regulated by miRNAs [7,8], thereby influencing several different pathways and processes in the cell, including development, differentiation, apoptosis, and cell proliferation [9-11].

As miRNAs are involved in fine-tuning gene expression in the cell [1,2], deregulation of miRNA expression could lead to altered gene expression, which might contribute to the development of cancer [12]. Several studies have shown a differential miRNA-expression profile in cancer as compared with normal controls [13-15]. Although specific miRNAs can be upregulated in cancer [16], global miRNA downregulation is a common trait of human malignancies [13,17]. Furthermore, miRNAs are involved in the metastatic cascade, which is the most dismal feature of tumor biology with respect to patient prognosis.

MiRNA-expression profiling of primary tumor samples and their associated metastases identified both prometastatic and metastasis-suppressor miRNAs [15]. These miRNAs modulate the expression of metastasis-associated genes [18,19], both directly and indirectly, by influencing the epigenetic machinery [20].

Breast cancer is the most frequent carcinoma and the second most common cause of cancer-related mortality in women [21]. In the past decade, it has been repeatedly shown that breast cancer is a heterogeneous condition consisting of at least five [22] but possibly more [23,24] molecular subtypes. These molecular subtypes (Luminal A, Luminal B, Basal-like, ErbB2+, and Normal-like) are characterized by specific mRNA-expression profiles. Blenkiron and colleagues [14] showed that these specific mRNA-expression profiles are at least partially attributable to differential miRNA expression. Also, Iorio and colleagues [25] identified a global pattern of miRNA deregulation in breast cancer tissue when compared with normal breast tissue, hinting at the importance of miRNA deregulation in the development of breast cancer in general.

As miRNAs appear to be critical regulators of tumor biology, their potential as prognostic and predictive biomarkers has recently been given attention. In addition, their great stability when compared with mRNA molecules, both in blood samples and in formalin-fixed, paraffin-embedded tissue samples, offers a great advantage [26,27]. Levels of miRNAs do not substantially change when serum or plasma samples are subjected to freeze-thaw cycles, boiling, or maintenance at room temperature [28,29]. As the bloodstream is easily accessible, blood-borne miRNAs or circulating miRNAs hold the potential to serve as noninvasive biomarkers in oncology.

Recently, Heneghan and colleagues [30] showed that miRNA expression is detectable in whole blood, plasma, and the serum of cancer patients and healthy controls. In addition, miRNA-195 was identified as a potential biomarker for detecting noninvasive and early-stage breast disease [30].

The goal of this study was twofold. First, we aimed to identify patterns of miRNA deregulation in breast cancer. Therefore, we compare miRNA-expression patterns between breast tumor samples classified according to the molecular subtypes and between breast tumor samples and normal breast samples. We hypothesize that such profiles can be informative for breast cancer detection and prognosis and might assist in defining specific targets for future therapy.

Second, we investigated whether the expression levels of miRNAs are measurable in blood samples from patients with breast cancer and healthy volunteers and if such expression profiles are potentially useful for the detection and staging of breast cancer.

## Materials and methods

### Patients and samples collection

Tumor and blood samples were obtained from patients with breast adenocarcinoma treated in the Breast Clinic of the General Hospital Sint-Augustinus (Antwerp, Belgium). Tissue and serum samples were derived from two entirely independent populations. Each patient gave written informed consent. This study was approved by the Institutional Review Board. Clinicopathologic data are stored in a database in accordance with hospital privacy rules and are summarized in Table 1. All tissue samples were stored in liquid nitrogen within 15 minutes after excision (median delay of 9 minutes). Healthy control tissue was obtained from breast-reductive surgery. None of the control samples showed pathologic changes. In total, 84 tumor samples and eight healthy control samples were included.

**Table 1:** Clinicopathologic data

Parameter	Group	Tissue (*n *= 84)	Serum (*n *= 75)
T status	1	27 (32%)	31 (41%)
	2	27 (32%)	22 (29%)
	3	6 (7%)	4 (5%)
	4	24 (29%)	18 (25%)
N status	0	35 (42%)	28 (37%)
	1	21 (25%)	15 (20%)
	2	14 (17%)	10 (13%)
	3	13 (15%)	9 (12%)
	4	1 (1%)	13 (18%)
M status	0	70 (83%)	4 (5%)
	1	14 (17%)	71 (95%)
ER status	Negative	25 (30%)	23 (31%)
	Positive	59 (70%)	52 (69%)
PR status	Negative	45 (54%)	36 (48%)
	Positive	39 (46%)	39 (52%)
ErbB2 status	Negative	62 (74%)	49 (65%)
	Positive	22 (26%)	26 (35%)
Grade	1	9 (11%)	14 (19%)
	2	32 (38%)	29 (39%)
	3	43 (51%)	32 (42%)
Stage	I	24 (29%)	19 (25%)
	II	20 (24%)	14 (19%)
	III	27 (32%)	22 (29%)
	IV	13 (15%)	20 (27%)
Disease status	Progressive	29 (35%)	47 (63%)
	Nonprogressive	55 (65%)	28 (37%)

ErbB2 status is determined by using the Hercep test with confirmation by FISH. Disease status is determined by using the RECIST criteria.

The collection of serum samples was described previously [31]. In brief, samples were prospectively obtained from 75 patients with breast cancer and 20 healthy volunteers. Patients were divided into three groups: four patients with localized breast cancer (group A), 55 patients with metastatic breast cancer receiving treatment (group B), and 16 patients with untreated metastatic breast cancer (group C). The blood samples of patients with metastatic disease were taken during the course of treatment. For all these samples, circulating tumor cells (CTCs) were enumerated by using the CellSearch system (Veridex, Warren, NJ, USA), CK19, and mammaglobin mRNA expression was recorded, the ADNAgen test for detection of CTCs was performed, and levels of total plasma DNA and serum methylated DNA for *ESR1*, *RASSF1A*, or *APC1 *were measured in earlier studies [31,32]. Disease status was assessed by using the RECIST (Response Evaluation Criteria in Solid Tumors) criteria without knowledge of the patient's CTC or circulating DNA results [33]. Stable disease was measured up to 8 weeks after the initiation of therapy. In addition, we collected blood samples from an additional series of 18 unselected patients to evaluate which blood medium (that is, serum, plasma, platelet-rich plasma, whole blood, or peripheral blood mononuclear cells (PBMCs)) was best suited for extraction of small RNAs (sRNAs).

### RNA extraction, cDNA synthesis, and miRNA quantification for tissue samples

After tissue disruption, total RNA was extracted by using the *mir*Vana miRNA Isolation Kit (Ambion, Austin, TX, USA) according to the manufacturer's instructions for total RNA isolation. In brief, the sample was homogenized in a denaturing lysis solution, followed by an acid-phenol:chloroform extraction. Thereafter, the sample was purified on a glass-fiber filter and quantified by using the Nanodrop ND1000 (NanoDrop Technologies, Waltham, MA, USA). Total RNA (100 ng) was converted to cDNA by priming with two pools of stem-looped RT primers (Megaplex RT Primers, Human Pool A & B; Applied Biosystems, Foster City, CA, USA) in combination with the TaqMan MicroRNA Reverse Transcription Kit (Applied Biosystems), allowing the simultaneous transcription of 377 unique miRNAs and six endogenous controls per primer pool. In brief, 3 μl of total RNA was supplemented with RT primer mix (×10), dNTPs with dTTP (100 m*M*), Multiscribe Reverse Transcriptase (50 U/μl), RT buffer (×10), MgCl_2 _(25 m*M*), and RNase inhibitor (20U/μl) in a total reaction volume of 7.5 μl.

Thermal-cycling conditions were as follows: 40 cycles at 16°C for 2 minutes, 42°C for 1 minute, and 50°C for 1 second, followed by reverse transcriptase inactivation at 85°C for 5 minutes. The Megaplex RT product (2.5 μl) was preamplified by using the TaqMan PreAmp Master Mix (Applied Biosystems) and preamplification primers in a 25-μl PCR reaction. For each pool of stem-looped RT primers in the cDNA reaction, a different pool of PreAmp Primers (Human Pool A & B; Applied Biosystems) was used. Thermal-cycling conditions were as follows: 95°C for 10 minutes, 55°C for 2 minutes, and 75°C for 2 minutes, followed by 12 cycles of 95°C for 15 seconds and 60°C for 4 minutes. MiRNA quantification was performed with the TaqMan Human MicroRNA Array sets A & B (Applied Biosystems), each containing 384 TaqMan miRNA assays. The PreAmp product was diluted fourfold. Each of the eight wells was loaded with 100 μl of PCR reaction mix, containing 50 μl of TaqMan Universal PCR Master Mix, no AmpErase uracil *N*-glycosylase (UNG) (Applied Biosystems), 1 μl of diluted PreAmp product, and 49 μl of nuclease-free water. Thermal-cycling conditions were as follows: 94.5°C for 10 minutes, followed by 40 cycles at 97°C for 30 seconds and 59.7°C for 1 minute. All PCR reactions were performed on a 7900HT Fast Real-Time PCR System (Applied Biosystems).

To test the efficiency of the miRNA assays, we compared the Ct-values of an undiluted sample with those of a 10-fold diluted sample. To evaluate the linearity of the preamplification, we compared the Ct-values of all miRNAs on both array cards for one sample before and after preamplification. The reproducibility of the arrays was tested by analyzing four samples in duplicate. The robustness of the TaqMan RT-PCR method was investigated by comparing the qRT-PCR miRNA expression profile of 12 samples with their miRNA expression profile obtained by using the nCounter Analysis System (Nanostring Technologies, Seattle, WA, USA). This system is a medium-high throughput gene-expression quantification system with PCR sensitivity that uses a novel digital technology based on direct multiplexed measurement of miRNA expression. Besides a direct quantification, the workflow incorporates only one enzymatic step (ligase step to enable tagging of the miRNAs) instead of three enzymatic steps in the qRT-PCR workflow, thereby substantially reducing the possibility for technical bias. The nCounter experiment was performed in collaboration with the VIB MicroArray Facility (O&N, UZ Gasthuisberg, Leuven, Belgium).

### RNA extraction, cDNA synthesis, and miRNA quantification for blood samples

First, we evaluated which blood medium was best suited for the extraction of sRNA molecules. Therefore, plasma, platelet-rich plasma, serum, whole blood, and PBMCs were obtained from 18 patients with breast cancer. Peripheral blood was collected in a 9-ml EDTA tube, from which 3 ml of whole blood was transferred into a cryovial while the remaining blood was centrifuged slowly (150 *g*, 20 minutes) at 4°C to generate platelet-rich plasma. Plasma and PBMCs were obtained in an 8-ml CPT tube, which was centrifuged (1,650 *g*, 20 minutes) at room temperature. Plasma and PBMC aliquots were transferred into separate cryovials. Finally, 8 ml blood was collected in serum separator tubes (Becton Dickinson, Franklin Lakes, NJ, USA) and centrifuged (2,000 *g*, 10 minutes) at room temperature. All samples were stored at -80°C until use.

sRNA was isolated from 200 μl of each medium by using the microRNA Isolation Kit (BioChain Institute Inc, Hayward, CA, USA) according to the manufacturer's instruction for sRNA purification. In brief, after adding lysis buffer to the sample for homogenization, 20 μl of Proteinase K solution (Qiagen, Valencia, CA, USA) was added and incubated for 10 minutes at 75°C to digest the excess of proteins released after addition of the lysis buffer. This was followed by an acid-phenol:chloroform extraction. Small and large RNAs were separated by using a centrifugation step, after which the large RNAs were retained on a glass-fiber filter. The sRNA molecules were recovered from the flow-through by purifying them on a second glass-fiber filter, and their concentration and purity (A260/A280 and A260/A230) was recorded by using the NanoDrop ND1000 (NanoDrop Technologies, Waltham, MA, USA). The concentrations were compared by using a Kruskal-Wallis test with Tukey HSD *post hoc *testing.

To evaluate circulating miRNA expression in blood samples from 20 healthy volunteers and 75 patients with breast cancer, we isolated total RNA, as described before. Isolated total RNA was reverse transcribed to produce cDNA by using the TaqMan MicroRNA Reverse Transcription Kit (Applied Biosystems) by priming with TaqMan MicroRNA Assays (Applied Biosystems) directed at 4 miRNAs identified by comparing tumor tissue with normal breast tissue (*vide supra*). In addition, miR-16 expression was determined as a normalization factor. In brief, each 15-μl reaction contained 0.15 μl 100 m*M *dNTPs with dTTP, 1.0 μl Multiscribe Reverse Transcriptase (50 U/μl), 1.50 μl RT Buffer (×10), 0.19 μl RNase Inhibitor (20 U/μl), 4.16 μl nuclease-free water, 5.0 μl total RNA, and 3.0 μl RT primer. Thermal-cycling conditions were 30 minutes at 16°C, 30 minutes at 42°C, and 5 minutes at 85°C. Each 20-μl reaction for the real-time quantitative PCR contained 1.0 μl real-time primer, 1.33 μl product from RT reaction, 10.0 μl TaqMan Universal PCR Master Mix, no AmpErase UNG (Applied Biosystems), and 7.67 μl nuclease-free water. The reactions were performed in duplicate on a 7900HT Fast Real-Time PCR System in the 9600 emulation mode, with conditions of 10 minutes at 95°C, followed by 40 cycles of 15 seconds at 95°C and 1 minute at 60°C. The mirVana miRNA Reference Panel (Ambion, Austin, TX, USA) was included in each PCR plate in a 2,000-fold dilution to correct for between-plate differences.

### Statistics and bioinformatics

All subsequent analyses were performed by using BioConductor in R. To reduce technical variation, the miRNA assays with a PCR efficiency outside the range of 2log(10) or 3.32 ± 25% [34] and those with Ct values above 35 in at least 25% of the cases were filtered out. By using efficient and informative miRNA assays only, we calculated the mean difference between the Ct values of one sample before and after preamplification. To avoid technical bias, we excluded miRNA assays with a difference in Ct values before and after preamplification outside the range of the mean value ± 25%. For the final set of miRNAs, we calculated the mean expression level per sample and used this value as a normalization factor to account for differences in input material [35]. Relative miRNA expression levels were calculated by using the ΔCt-method [36] and log2-transformed to obtain a normal distribution. To investigate assay reproducibility, we correlated the expression profiles of the duplicate samples by using the Spearman correlation coefficient.

An additional technical validation was done by performing a pairwise correlation analysis between the miRNA profiles obtained by qRT-PCR and the nCounter Analysis System for the 12 samples analyzed on both platforms. Both correlation analyses were done by using the normalized expression profiles of the 327 common miRNAs only.

Unsupervised hierarchical cluster analysis (UHCA), with the Manhattan distance as similarity metric and Ward clustering as the dendrogram drawing method, was performed to visualize global themes in the expression data. We classified samples according to the miRNA-centroids for molecular subtypes published by Blenkiron *et al. *[14]. Therefore, we correlated the molecular subtype-specific miRNA-expression profiles of each sample with each of the five miRNA-based expression centroids by using the Spearman correlation coefficient.

The resulting classification was compared with the UHCA result. For 66 samples with available Affymetrix-profiles, we compared the correlation coefficients between the samples grouped according to the SSP (single-sample predictor)-defined molecular subtype classification [37] obtained through mRNA-expression profiling reported in earlier studies [38,39]. Significance was assessed by using the Mann-Whitney *U *tests.

Next, we aimed to identify molecular subtype-specific miRNAs. Therefore, we performed a pairwise comparison of the different molecular subtypes, defined through mRNA-expression profiling, by using regression analysis with the limma-package. False Discovery Rate (FDR) correction was performed by using the Benjamini and Hochberg step-up procedure. For each subtype, we crossed the lists of differentially expressed miRNAs resulting from the pairwise comparisons involving the desired subtype in search for common miRNAs.

By using regression analysis, we identified differentially expressed miRNAs between normal and tumor samples. Resulting *P *values were corrected for false discovery, as described earlier. To investigate global over- or underexpression in normal samples, we calculated the median expression level of the differentially expressed miRNAs per sample. These median expression values were compared by using Mann-Whitney *U *testing. The top four differentially expressed miRNAs by fold-change were selected for further analysis. For these miRNAs, we identified target mRNAs in at least two of three public databases (PicTar, TargetScan, and Miranda) by using the RmiR-package. These target-gene lists were subjected to Ingenuity Pathway Analysis (IPA) to study the implications of the identified miRNAs in cancer biology.

Expression levels of circulating miRNAs were calculated with miR-16 as normalization factor. Raw Ct values measured in the miRNA Reference Panel were subtracted from the Ct values measured in the samples, yielding a between-plate corrected expression value for each miRNA per 200 μl of serum. The miR-16 normalized expression value was calculated by subtracting the between-plate corrected expression value for miR-16 from the between-plate corrected expression values for the remaining miRNAs (ΔΔCt-method). Relative expression values were calculated by using the 2^-ΔΔCt ^method [36]. To compare the expression data with categoric variables, the Mann-Whitney *U *test was performed. To compare expression data with continuous variables, Spearman correlation coefficients were calculated.

## Results

### Technical validation of miRNA profiling in tissue samples

**Figure 1:**
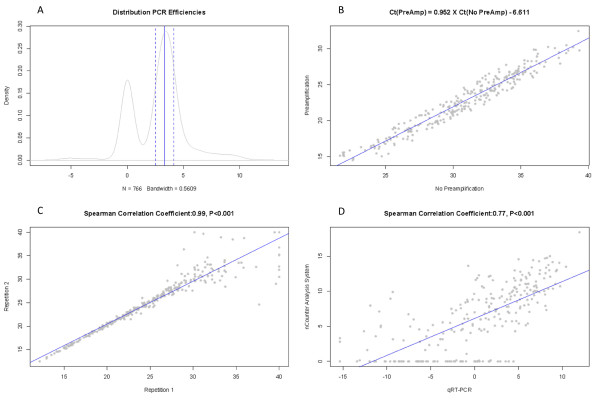
**The distribution of polymerase chain reaction (PCR) efficiencies, calculated as the differences between the Ct values of undiluted sample and the Ct values of a 10-fold diluted sample**. **(A) **Theoretically, this difference should equal 3.32 or 2log(10). All miRNA assays with a difference in Ct value between 3.32% and 25% were included for further analysis. A blue dashed line indicates the boundaries of the interval; a blue solid line indicates the theoretical expected value of 3.32. To account for differences in preamplification, we compared the Ct values of a sample before and after preamplification. The median difference in Ct value was 8, and all miRNA assays with a difference of 8% ± 25% were included for further analysis. The scatterplot in **(B) **demonstrates an almost perfect linear relation for those selected miRNAs before and after preamplification. The blue line represents the regression line, for which the equation is given on top of the scatterplot. To evaluate assay reproducibility, we tested four samples in duplicate. The scatterplot in **(C) **demonstrates the result for one of these samples. The blue line represents the regression line, and the correlation coefficient resulting from the comparison of both profiles is given on top of the scatterplot. Further technical validation of our miRNA-expression data was performed for 12 samples by analyzing their miRNA-expression profile with the nCounter Analysis System and comparing this result with the qRT-PCR-based miRNA-expression profile. The scatterplot in **(D) **demonstrates the result for one of these samples. The blue line represents the regression line, and the correlation coefficient resulting from the comparison of both profiles is given on top of the scatterplot.

First, we excluded 292 miRNA assays (A panel, 83; B panel, 209) with a Ct value above 35 in at least 25% of the samples, leading to 462 informative miRNAs (A panel, 294; B panel, 168). Before performing the data normalization, we checked the PCR efficiencies of all miRNA assays on the array cards by performing a 10-fold dilution series and subtracting the Ct values of the undiluted sample from the Ct values of the diluted sample. Theoretically, for an efficient PCR reaction, this difference should equal 2log(10) or 3.32. We excluded 23 (A panel, 14; B panel, 9) miRNA assays with PCR-efficiencies outside the range of 3.32 ± 25%. The distribution of the PCR efficiencies and the cut-off values for exclusion are shown in Figure 1A.

Next we evaluated the linearity of the preamplification by comparing the miRNA-expression profiles of a sample before and after preamplification. This analysis was done for 439 miRNAs that remained after exclusion of noninformative and inefficient miRNA assays. The mean difference between the Ct values before and after preamplification was 8, and miRNA assays with a difference in Ct value outside the range of 8 ± 25% were excluded from further analysis (A panel, 30; B panel, 36). As such, the final data set consisted of 373 miRNAs that were normalized by using the ΔCt method with the median Ct value per sample as normalization factor. The scatterplot comparing the Ct values for those 373 miRNAs before and after preamplification is shown in Figure 1B, and regression analysis demonstrated a significant and linear relation (*R^2 ^*= 0.927; *P *< 0.001).

**Figure 2:**
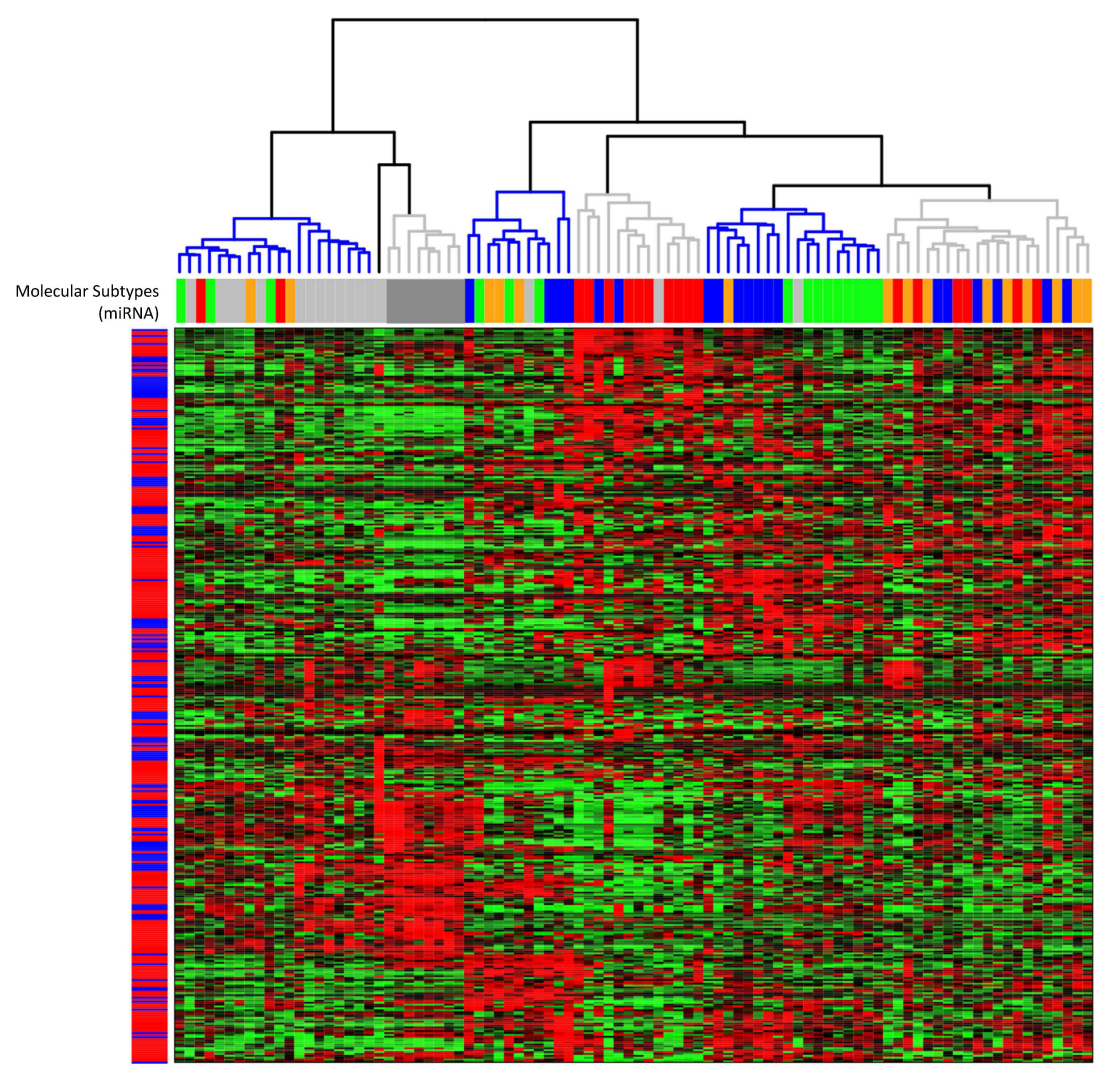
**Heatmap showing the result of an UHCA (Manhattan distance, Ward linkage) for all 373 miRNAs in all 92 samples**. The miRNA-expression data are represented in matrix format, with rows indicating miRNAs and columns indicating samples. Overexpressed miRNAs are color-coded red, and repressed miRNAs are color-coded green. Color saturation indicates the level of overexpression. Six samples clusters could be discerned based on miRNA-expression differences, indicated by alternating blue and grey colors in the dendrogram. Underneath the sample dendrogram, the molecular-subtype classification is indicated (red, Basal-like; orange, ErbB2+; green, Luminal A; blue, Luminal B; gray, Normal-like). The true-normal breast samples are indicated by a darker shade of gray. The colored bar to the side of the heatmap indicates the array card to which the corresponding assay is allocated (red, A; blue, B).

Next, we investigated the effect of profiling miRNA expression by using two different array cards (A and B) per sample. Therefore, UHCA was performed on the normalized miRNA expression data, and the result is shown in Figure 2. The cluster pattern of the miRNAs, represented by the dendrogram in the Y-axis, reveals that the assays allocated at different array cards are not segregated. In addition, miRNA assays directed at different isoforms of the same miRNA and represented on different array cards cluster on terminal branches in approximately 80% of the cases. These data indicate that variation in miRNA expression related to the incorporation of two separate assays per sample is minor.

Finally, we evaluated the reproducibility of the miRNA assays. Therefore, we analyzed four samples in duplicate and compared their normalized miRNA expression profiles. A representative scatterplot is show in Figure 1C, and all scatterplots are shown in Additional file 1. Spearman correlation coefficients ranged from 0.98 to 0.99 (all *P *values < 0.001), indicating good assay reproducibility.

Next, we analyzed the miRNA expression profiles of 12 samples by using the nCounter Analysis System and compared them with the normalized expression data obtained through qRT-PCR. The Spearman correlation coefficients ranged from 0.63 to 0.75 with a median value of 0.72 (all *P *values < 0.001). Scatterplots for all comparisons are shown in Additional file 2, and a representative scatterplot is provided in Figure 1D.

Overall, our data indicate that technical variation in expression for the panel of 373 miRNAs is minor.

### miRNA expression profiling of breast tumor and normal breast samples

With the miRNA centroids for molecular-subtype classification [14], we classified the 84 breast cancer tissue samples and eight normal tissue samples in our data set. 18, 15, 15, 19, and 25 samples were classified as Basal-like, ErbB2+, Luminal A, Luminal B, and Normal-like, respectively. The classification result is shown under the dendrogram in Figure 2. We observed a significant (*P *< 0.001) agreement between the miRNA-based molecular subtype classification and the clustering pattern of the tissue samples after UHCA. Downstream of the first bifurcation, we observe a cluster composed of 76% of Normal-like samples, which was further divided into two clusters separating the Normal-like tumor samples from the normal breast samples. Further division of the dendrogram yielded a cluster composed of 77% of Basal-like samples and a cluster enriched for Luminal samples (that is, 89% of the samples classify as Luminal A or Luminal B). In addition, 60% of the ErbB2+ samples fell into one sample cluster. Interestingly, downstream of the third bifurcation, we observed a sample cluster not enriched for any of the molecular subtypes (Luminal A, 3; Luminal B, 4; ErbB2+, 3; and Normal-like, 1). Given the hierarchy of the dendrogram, this sample cluster may well represent a novel miRNA-based breast cancer subtype. Overexpressed miRNAs in this sample group are known for their tumor-suppressive roles in (breast) cancer: the miR-200 family (miR-200a, miR-200b, miR-200c, and miR-141), the let-7 family (let-7a, let-7f, and let-7g), and NFκB-regulating miRNAs (miR-146a and miR-155).

**Figure 3:**
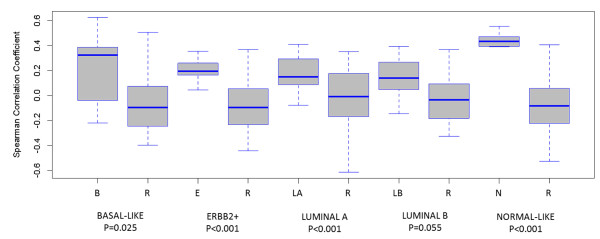
**Comparison of the mRNA-based molecular subtype classification by using the SSP method with the miRNA-based classification by using the expression centroids reported by Blenkiron and colleagues**. This analysis was performed only for those samples for which Affymetrix mRNA-expression profiles are available (*N *= 66). The SSP-classification is provided in the X-axis (B, Basal; E, ErbB2+; LA, Luminal A; LB, Luminal B; N, Normal-like; and R, Rest). The Spearman correlation coefficients resulting from the miRNA-based molecular subtype classification are indicated in the Y-axis. For each miRNA-based molecular subtype-specific centroid and each sample in our data set, the Spearman correlation coefficients were determined. The molecular subtype-specific correlation coefficients were statistically compared between samples belonging to and not belonging to the SSP-defined molecular subtype of interest. *P *values are indicated under the corresponding boxplots.

For 66 of 92 samples, Affymetrix mRNA expression profiles were obtained in previous studies [38,39]. Classification of these samples according to the SSP-algorithm yielded an agreement of 66% with the classification according to the miRNA centroids. Subtype-specific correlation coefficients were compared between the SSP-defined molecular subtypes, and results are shown in Figure 3. For all comparisons, the subtype-specific correlation coefficients obtained by using the miRNA-centroids were significantly elevated in the group of samples classified in the corresponding subtype by using mRNA data (all *P *values < 0.05). When dichotomizing the Spearman correlation coefficients per subtype relative to 0, we observed an average classification error rate of 36%, with the highest and lowest classification error rates observed for the Luminal B and ErbB2+ samples (44% and 27%), respectively.

To identify subtype-specific miRNAs, we performed pairwise comparisons between tumor samples grouped by the SSP-defined molecular subtypes. The results are summarized in Table 2. At a *P*-value cut-off level of 0.01 (maximal FDR of 15%), we identified 16, 0, 2, 3, and 40 miRNAs specific for the Basal-like, ErbB2+, Luminal A, Luminal B, and Normal-like subtypes, respectively. Comparison of these results with the expression data published by Blenkiron and colleagues [14] revealed remarkably similar expression patterns for several key miRNAs. For example, miR-135b and miR-106a are upregulated in Basal-like breast cancers in both studies. Also, miR-100 and miR-145 show comparable expression patterns in both studies, with elevated expression in the Normal-like and Luminal samples. Detailed results are provided in Additional file 3.

**Table 2:** Identification of subtype-specific miRNAs

Subtype	Comparator	Number	Common
Basal-like	ErbB2+	26	hsa-miR-135b#, hsa-miR-135b, hsa-miR-934, hsa-miR-577, hsa-miR-501-5p, hsa-miR18a#, hsa-miR-92a, hsa-miR-106a, hsa-miR-17, hsa-miR-18b, hsa-miR-18a, hsa-miR-20a, hsa-miR-17#, hsa-miR-15b#, hsa-miR-19a, hsa-miR-500
	Luminal A	53	
	Luminal B	33	
	Normal-like	90	
			
ErbB2+	Basal-like	26	-
	Luminal A	25	
	Luminal B	14	
	Normal-like	107	
			
Luminal A	Basal-like	53	hsa-miR-148a, hsa-miR-219-5p
	ErbB2+	25	
	Luminal B	15	
	Normal-like	106	
			
Luminal B	Basal-like	33	hsa-miR-30d#, hsa-miR-30d, hsa-miR-342-3p
	ErbB2+	14	
	Luminal A	15	
	Normal-like	109	
			
Normal-like	Basal-like	90	hsa-miR-136#, hsa-miR-497, hsa-miR-139-5p, hsa-miR-99a#, hsa-miR-145#, hsa-miR-195, hsa-miR-143, hsa-miR-145, hsa-miR-335, hsa-miR-125b-2#, hsa-miR-139-3p, hsa-miR-7-2#, hsa-miR-216b, hsa-miR-487b, hsa-miR-100, hsa-miR-410, hsa-miR-204, hsa-miR-376a, hsa-miR-99a, hsa-miR-337-3p, hsa-miR-27a#, hsa-miR-411, hsa-miR-656, hsa-miR-495, hsa-miR-551b#, hsa-miR-770-5p, hsa-let-7b#, hsa-miR-378, hsa-miR-215, hsa-miR-127-3p, hsa-let-7c#, hsa-miR-379, hsa-miR-422a, hsa-miR432, hsa-miR-299-5p, hsa-miR-494, hsa-miR-378, hsa-miR-511, hsa-miR-23a#, hsa-miR-452
	ErbB2+	107	
	Luminal A	106	
	Luminal B	109	

P values, < 0.01; maximal false discovery rate, 15%.

**Figure 4:**
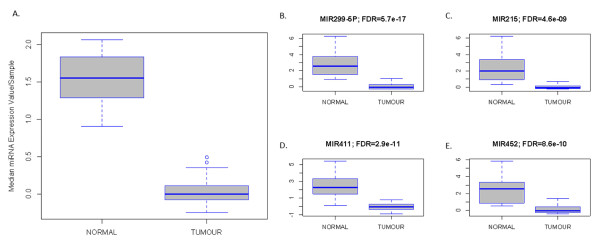
**Comparison of normal breast samples with tumor samples**. We identified 59 differentially expressed miRNAs between tumor samples and normal breast samples. The median expression of these miRNAs is significantly elevated in normal breast samples, as illustrated by the boxplot **(A)**. The top four miRNAs (miR-215, miR-299-5p, miR-411, and miR-452) with the greatest difference between normal breast samples and breast tumor samples by fold change are depicted in panels **B **through **E**. The corresponding false discovery rate is provided in top of each boxplot. All miRNAs are significantly overexpressed in normal breast samples.

Finally, we compared the miRNA expression profiles of tumor samples with the normal breast samples obtained after breast-reductive surgery. As reported earlier, the clustering pattern of the tissue samples (Figure 2) suggests major differences in miRNA expression between the tumor samples and normal breast samples. We identified 59 differentially expressed miRNAs at an FDR less than 1%. The median expression value of these miRNA per sample was significantly higher in normal breast samples (Normal, 1.542; Tumor, 0.024; *P *< 0.001). Data are presented in boxplot format in Figure 4A. The top four miRNAs by fold-change (miR-299-5p, miR-215, miR-411, and miR-452) were selected as potential biomarkers for breast cancer detection (Figure 4B through 4E). With the RmiR-package, we identified 611, 715, 575, and 1,863 mRNA targets for the set of selected miRNAs, respectively, which were analyzed by using Ingenuity Pathway Analysis. For each miRNA, the five most relevant networks with their most strongly enriched molecular and cellular functions (*P *< 0.001) are listed in Table 3. Comparative analysis of enrichment patterns demonstrated that all miRNAs were involved in the regulation of global oncogenic processes like cell proliferation, cell death, and cellular movement.

**Table 3:** Biologic and cellular functions of miR-215, miR-299-5p, miR-411, and miR-452

miRNA	Network ID	Score	Number of genes	Top associated functions
miR-215	1	37	28	Tissue morphology, cell death, drug metabolism
	2	20	19	Developmental disorder, gene expression, genetic disorder
	3	20	19	Cellular movement, immune cell trafficking, skeletal and muscular system development and function
	4	14	15	RNA damage and repair, cell death, molecular transport
	5	13	14	Genetic disorder, cellular assembly and organization, cellular function and maintenance
				
miR-299-5p	1	35	29	Cell-to-cell signaling and interaction, cellular growth and proliferation, tumor morphology
	2	24	23	Gene expression, cellular movement, lipid metabolism
	3	13	16	Cellular assembly and organization, DNA replication, recombination, and repair, gene expression
	4	13	16	Cell morphology, cellular development, protein synthesis
	5	11	14	Cell death, renal necrosis/cell death, cellular compromise
				
miR-411	1	33	26	Cell death, cell-to-cell signaling and interaction, cell-mediated immune response
	2	27	23	Cardiovascular system development and function, organ development, organismal development
	3	17	17	Gene expression, protein synthesis, antimicrobial response
	4	14	15	Cell death, cellular growth and proliferation, cellular assembly and organization
	5	11	13	Inflammatory response, dermatologic diseases and conditions, inflammatory disease
				
miR-452	1	31	32	Gene expression, cellular movement, cell death
	2	27	30	Cell-to-cell signaling and interaction, connective tissue development and function, cell morphology
	3	25	29	Cellular growth and proliferation, inflammatory response, cell death
	4	25	29	Cellular development, gene expression, nervous system development and function
	5	25	29	Cellular growth and proliferation, cardiovascular disease, tissue morphology

### Circulating miRNA expression

To evaluate which blood medium was best suited for investigating miRNA expression, we extracted sRNA molecules from serum, plasma, platelet-rich plasma, whole blood, and PBMCs. A significant increase in sRNA concentration was observed only when comparing the results obtained in whole blood with the results obtained in other media (Kruskal-Wallis, *P *< 0.001; Tukey HSD *post hoc*, all *P *values < 0.001). Results are shown in Additional file 4. As our aim was to measure circulating, tumor-specific miRNA expression, we decided not to perform subsequent analyses on platelet-rich plasma, whole blood, or PBMCs because of the possible contamination of host-specific miRNA expression. Given a slight, not significant, increase in sRNA concentration in serum when compared with plasma (5.3 μg/ml versus 4.2 μg/ml), in addition to a more-consistent sRNA yield in serum (CV_serum_, 50.4%; CV_plasma_, 94.5%), we decided to use serum to evaluate circulating miRNA expression.

**Figure 5:**
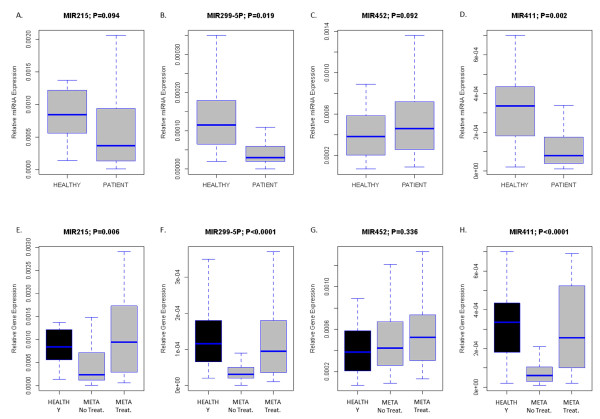
**Comparison of the expression profiles of miR-215, miR-299-5p, miR-411, and miR-452 between serum samples from patients with breast cancer and serum samples from healthy volunteers (A through D)**. The boxplots on panels **E **through **H **represent the comparison of the expression profiles of the same miRNAs between serum samples from healthy volunteers and from patients with metastatic breast cancer receiving and not receiving treatment. The *P *values indicating the significance of the difference are indicated on top of the boxplots.

The expression of four miRNAs (miR-299-5p, miR-215, miR-411, and miR-452) with the greatest fold-change, when comparing normal breast tissue with breast tumor samples, was analyzed in serum samples from 75 patients with breast cancer and 20 healthy volunteers. We observed higher expression values for all investigated miRNAs, except for miR-452, in serum from healthy volunteers. Significant (*P *< 0.05) values were obtained for miR-299-5p and miR-411, whereas trends (*P *< 0.10) were observed for miR-215 and miR-452. Results are shown in Figure 5.

We next compared the expression levels of miR-215, miR-299-5p, miR-411, and miR-452 in serum from patients with metastatic breast cancer receiving treatment (group B), patients with untreated metastatic breast cancer (group C), and healthy volunteers. The group of patients with localized breast cancer was not included in this analysis because of low sample size (*n *= 4). Results are shown in Figure 5. Kruskal-Wallis testing revealed significant (*P *< 0.05) between-group differences for all miRNAs, except miR-452. Tukey HSD *post hoc *testing revealed that the lowest expression values were observed in patients with metastatic breast cancer, whereas expression levels returned to normal with treatment.

Finally, we compared the expression levels of the circulating miRNAs with clinicopathologic variables, response to treatment evaluated by the RECIST-criteria, presence of circulating tumor markers, and the presence of circulating methylated markers. These analyses were done for all samples except those derived from healthy volunteers. Results are shown in Table 4. Overall, few significant associations were observed. The expression levels for three of four miRNAs (miR-215, miR-299-5p, and miR-411) show a negative association with patient age at diagnosis (all *P *values < 0.100). Interestingly, all miRNAs have higher expression levels in serum from patients with progressive disease under treatment and for two of four miRNAs (miR-215 and miR-411); these differences were significant (*P *< 0.050). No associations between circulating miRNA expression and the presence of CTCs were observed. For miR-215 and miR-452, we observed positive associations (*P *< 0.05) between their expression levels in serum and the number of methylated genes (any combination of *ESR1*, *APC*, and *RASSF1A*) detected in plasma.

**Table 4:** Associations between circulating miRNA expression and clinicopathologic variables

Group	Variable	Test	MIR-215		MIR-299-5p		MIR-411		MIR-452	
			Result	*P-*value	Result	*P*-value	Result	*P-*value	Result	*P*-value
Clinicopathologic	Age	Spearman correlation	*R *= -0.184	***P *= 0.113**	*R *= -0.455	***P *< 0.001**	*R *= -0.362	***P *= 0.001**	*R *= -0.052	*P *= 0.657
	ER^a^	Mann-Whitney *U *test	*T *= -1.059	*P *= 0.295	*T *= 0.658	*P *= 0.516	*T *= 0.305	*P *= 0.763	*T *= 1.194	*P *= 0.244
	PR^a^	Mann-Whitney *U *test	*T *= -0.672	*P *= 0.504	*T *= -0.193	*P *= 0.848	*T *= -0.645	*P *= 0.521	*T *= 1.519	*P *= 0.136
	HR^a^	Mann-Whitney *U *test	*T *= -1.233	*P *= 0.225	*T *= 0.632	*P *= 0.534	*T *= 0.299	*P *= 0.767	*T *= 1.015	*P *= 0.321
	ERBB2^a^	Mann-Whitney *U *test	*T *= 0.051	*P *= 0.960	*T *= -1.237	*P *= 0.222	*T *= -1.594	*P *= 0.120	*T *= -0.841	*P *= 0.406
	TNBC^a^	Mann-Whitney *U *test	*T *= -0.399	*P *= 0.699	*T *= -0.310	*P *= 0.765	*T *= -0.365	*P *= 0.725	*T *= 0.281	*P *= 0.785
	P53^a^	Mann-Whitney *U *test	*T *= 0.256	*P *= 0.800	*T *= 1.041	*P *= 0.304	*T *= 1.617	***P *= 0.113**	*T *= -0.953	*P *= 0.347
										
RECIST	Progressive disease^a^	Mann-Whitney *U *test	*T *= -2.404	***P *= 0.019**	*T *= -0.998	*P *= 0.322	*T *= -2.488	***P *= 0.016**	T = -0.399	*P *= 0.691
										
Circulating markers	Number CTCs	Spearman correlation	*R *= 0.056	*P *= 0.635	*R *= -0.026	*P *= 0.826	*R *= 0.053	*P *= 0.655	*R *= -0.094	*P *= 0.424
	ADNAGen^a^	Mann-Whitney *U *test	*T *= -0.534	*P *= 0.596	*T *= 1.789	***P *= 0.078**	*T *= 0.782	*P *= 0.437	*T *= -2.144	***P *= 0.041**
	Mammaglobin expression	Spearman correlation	*R *= 0.047	*P *= 0.697	*R *= 0.005	*P *= 0.969	*R *= 0.086	*P *= 0.474	*R *= -0.107	*P *= 0.374
	Cytokeratin 19 expression	Spearman correlation	*R *= 0.191	***P *= 0.110**	*R *= -0.099	*P *= 0.409	*R *= -0.010	*P *= 0.933	*R *= 0.004	*P *= 0.968
	Plasma DNA concentration	Spearman correlation	*R *= 0.200	***P *= 0.085**	*R *= -0.100	*P *= 0.392	*R *= -0.043	*P *= 0.712	*R *= 0.085	*P *= 0.467
										
Methylated markers	RASSF1A	Spearman correlation	*R *= -0.129	*P *= 0.271	*R *= -0.198	*P *= 0.089	*R *= -0.189	***P *= 0.104**	*R *= 0.025	*P *= 0.829
	APC	Spearman correlation	*R *= -0.144	*P *= 0.217	*R *= -0.134	*P *= 0.253	*R *= -0.114	*P *= 0.332	*R *= -0.010	*P *= 0.930
	ESR1	Spearman correlation	*R *= 0.148	*P *= 0.205	*R *= 0.001	*P *= 0.997	*R *= 0.086	*P *= 0.461	*R *= 0.252	***P *= 0.029**
	Number methylated genes	Spearman correlation	*R *= 0.250	***P *= 0.031**	*R *= -0.159	*P *= 0.172	*R *= -0.039	*P *= 0.740	*R *= 0.262	***P *= 0.023**
	Methylation status^a^	Mann-Whitney *U *test	*T *= -1.583	***P *= 0.118**	*T *= 1.456	*P *= 0.152	*T *= 0.538	*P *= 0.593	*T *= -1.270	*P *= 0.208

^a^A negative *T*-value indicates a higher expression in the "positive" group.

## Discussion

We attempted to identify a panel of deregulated miRNAs in breast cancer and investigated their potential as biomarkers for the detection and staging of breast cancer by using blood-based testing. Before analyzing the miRNA-expression data, we first evaluated the performance of the PCR technology used throughout this study. To reduce the technical variation in our data set, we included only informative miRNAs assays (Ct values smaller than 35 in at least 25% of the cases) with similar PCR efficiencies and similar differences in Ct values before and after preamplification. The boundaries for PCR efficiency were defined as described in earlier studies [34], and the boundaries for preamplification efficiency were set alike. The expression data recorded by the final set of 373 selected miRNAs proved to be reproducible, and no between-array card difference was observed. Moreover, we noticed an above-moderate agreement between the qRT-PCR-based miRNA profiles of 12 samples with the miRNA profiles measured by using the nCounter Analysis System.

This is important for two reasons. First, the nCounter Analysis System incorporates only one enzymatic step (that is, a ligase treatment for attachment of the reporter tags) in its workflow and is therefore less prone to technical bias than is the PCR-based protocol that incorporates three enzymatic steps.

A second reason for comparing the miRNA expression profiles by using alternative profiling techniques is related to the fact that good normalization procedures for miRNA expression data are currently still lacking. The qRT-PCR-based miRNA-expression data in this study were normalized relative to the mean expression value of all miRNAs per sample, as proposed by Mestdagh *et al. *[35]. However, we think that this normalization procedure might have a major drawback because of the role of DICER1, a miRNA-preprocessing enzyme, in breast cancer. Recent reports have shown that the expression of DICER1 is different across the different molecular subtypes [14,40-42]. As DICER1 is involved in cleaving the precursor miRNAs into mature miRNAs, variation in DICER1 expression might result in altered turnover rates of the precursor miRNAs and, hence, higher concentrations of mature miRNAs in those tumor samples with higher DICER1 expression. Therefore, we reason that the mean miRNA-expression levels can vary depending on DICER1 expression and that normalization relative to the mean miRNA-expression level might obscure between-sample differences, particularly in breast cancer. The alternative approach would be to use the reference miRNAs provided on the array cards or miR-16, which is often suggested as reference miRNA. However, the CVs for these reference miRNAs were about threefold higher than the CV of the mean miRNA expression level per sample. In addition, about 20% of the miRNA assays on both array cards yielded more-robust expression data (data not shown).

Therefore, in spite of our previously raised concerns, we decided to normalize our expression data relative to the mean Ct value per sample and compare the results with the data obtained by using nCounter Analysis System, which uses a panel of five mRNA assays (RPLPO, RPL19, ACTB, B2M, and GAPDH) for data normalization. The above-moderate agreement between the miRNA-expression data obtained by using both profiling techniques lends credit to the biologic validity of our qRT-PCR-based miRNA-expression profiles.

Further evidence that the applied normalization procedure did not obscure molecular subtype-specific differences is derived from the UHCA, which showed that the molecular subtypes govern global themes in our miRNA expression data set. Also, the miRNA-based molecular subtype classification is in agreement with the classification resulting from the application of a more-validated algorithm on mRNA data (SSP) [37]. For example, the comparison of the miRNA-based expression profile of SSP-defined Basal-like breast tumors with the miRNA-based expression centroid for Basal-like breast cancer results in more-elevated Spearman correlation coefficients than when compared with the results obtained for non-Basal-like breast tumor samples. Although the classification error rate was substantial, we must keep in mind that the miRNA-based expression centroids reported by Blenkiron and colleagues [14] are based on a limited series of samples. Therefore, it is arguable that the expression centroids are not very stable, which affects the classification accuracy. When performing a supervised analysis, we were able to identify sets of specific miRNAs for each molecular subtype, except for the ErbB2+ breast tumor samples. Overall, our results are in line with previously reported data [14,43-45], except for the results with respect to the ErbB2+ subtype, for which an miRNA signature has been defined in the past [45]. Of note is the concordant overexpression of miRNAs belonging to the polycistronic miR-17-92 cluster (miR-17, miR-18a, miR-19a, miR-20a, and miR-92) and its paralogs (miR-18b and miR-106a) in Basal-like breast tumors. The miR-17-92 cluster is known to downregulate ERα in a MYC-dependent manner and inhibits the protein translation of AIB1, an ERα transcriptional coactivator [46]. Also, the miR-17-92 cluster is known to regulate cell migration, invasion, and metastasis in breast cancer by regulating ROCK [47] and the HBP1/β-catenin pathway [48].

Although the sample-clustering pattern based on the expression of 373 miRNAs demonstrated that the global themes in our expression data set are related to the presence of the classic molecular subtypes in breast cancer, we did identify one sample cluster without any connection to the classic molecular subtypes. This sample cluster originated early in the dendrogram, indicative of a specific miRNA-expression profile. Indeed, the heatmap did reveal an miRNA cluster, including members of the miR-200 family, members of the let-7 family, and NFκB-regulating miRNAs [49], that is overexpressed in this group of tumor samples, at a level exceeding the expression level observed in the Luminal-like sample cluster. The latter observation is at least remarkable, as all these miRNA families are known to inhibit stem cell-specific pathways, epithelial-to-mesenchymal transition, cell proliferation, and other global oncogenic processes [16,50-53]. Hence, their overexpression would induce a more-differentiated, less-proliferative, less-mesenchymal, and less-migratory/invasive cell phenotype. The presence of this tumor sample cluster with its particular molecular characteristics warrants further investigation. When focusing on the Normal-like samples, a clear and distinct miRNA profile was observed. In addition, the true normal breast samples constituted a coherent group inside the cluster of the Normal-like samples, suggesting vast differences in miRNA expression between tumor samples and normal breast samples. Indeed, supervised analysis revealed high numbers of differentially expressed miRNAs with nominal (uncorrected) *P *values less than 0.05.

The huge difference in miRNA expression between normal and tumor samples underlines the important role of miRNA deregulation in the development of breast cancer. After correction for false discovery, we observed that the majority of the differentially expressed miRNAs have attenuated expression levels in the tumor samples. The global repression of miRNAs in cancerous tissue relative to normal tissue has been reported previously and suggests that most miRNAs have a tumor-suppressive function [13]. This view is corroborated by reports on the cellular functions of the top four (miR-215, miR-299-5p, miR-411, and miR-452) differentially expressed miRNAs by fold-change. Song *et al. *[54] demonstrated that miR-215 overexpression in a colon cancer cell line reduced the proliferation rate and led to improved cell-cycle control, probably due to an increased expression of the cell-cycle control genes *p53 *and *p21*. Duan *et al. *[55] showed that miR-299-5p expression increased downstream of the tumor suppressor PRDM5 in HEK293 cells. In contrast, Fang *et al. *[56] showed that *SOX2*, a gene with tumor-promoting activity involved in cell proliferation and colony formation of LN229 glioblastoma multiforme cells, represses miR-452. The evidence for a role of miR-411 as tumor suppressor is less clear, but this miRNA is located at the 14q32.31 locus, which is known to harbor many tumor-suppressive miRNAs [57]. The biologic processes regulated by miR-215, miR-299-5p, miR-411, and miR-452, identified through the analysis of their respective target-gene lists, are in line with their role in maintaining cellular homeostasis.

Because of the marked overexpression of miR-215, miR-299-5p, miR-411, and miR-452 in normal breast samples, in addition to the fact that miRNAs have a proven stability in blood samples [29], we hypothesized that this panel of miRNAs might be suitable for the detection of breast cancer by using blood-borne testing. The reason for using serum samples for this purpose is twofold. First, we argued that miRNA-expression profiles in whole blood, platelet-rich plasma, and PBMCs would be dominated by host miRNA expression, and therefore would be less suitable for the detection of tumor-specific miRNA expression. Conversely, reports have shown that miRNA expression is also detectable in serum and plasma samples from healthy donors [58]. Second, we noticed a slightly higher and more consistent sRNA yield in serum as compared with plasma. When evaluating the relative expression profiles of miR-215, miR-299-5p, miR-411, and miR-452 in serum samples from patients with breast cancer and healthy volunteers, we recorded comparable expression profiles in tissue and blood samples, except for miR-452. Of note, when comparing absolute CT values, the expression differences between samples from patients with breast cancer and healthy volunteers were maintained, however, at a higher fold-change level. In addition, we observed that the reduction of miRNA expression was particularly obvious in serum samples from patients with untreated metastatic breast cancer, whereas the expression profiles "normalized" with treatment.

No associations between blood-borne miRNA expression in serum samples from patients with breast cancer and the classical clinicopathologic variables were observed, except for the patient's age at diagnosis. However, this should not be surprising, as our miRNA panel was not selected to make this distinction. Of note is the lack of associations between circulating miRNA expression and the presence of CTCs, measured by three alternative techniques. This observation suggests that recorded serum miRNA profiles are not CTC derived and that the mechanisms responsible for the release of miRNAs in the circulation are unrelated to the extravasation of tumor cells. Indeed, several reports have suggested that miRNAs are selectively released in the bloodstream by tumor cells either via exosomes or attached to lipoprotein complexes or within a functional RISC complex [59-63]. These mechanisms of secretion offer an explanation for the marked stability of miRNAs in the blood stream, due to shielding of the associated miRNAs from RNAse-activity. In addition, miRNAs secreted as such are functionally active and have been found to regulate gene expression in target cells, thereby providing alternative ways of cell-cell communication. This opens the possibility that miRNAs, secreted by tumor cells, evoke a response in host cells altering their expression profile, which explains how subtle differences in tumor-specific expression are measurable in a background of nontumorigenic expression. More specific in the context of our results, one could envision that tumor-driving miRNAs, secreted by tumor cells, affect the expression profile of host cells, which is reflected in the serum profile of breast cancer patients and explains the observed decrease in miRNA expression.

## Conclusions

The present data provide a technologically validated framework to elaborate on the study of miRNA-deregulation in the development of breast cancer. We potentially identified a novel subgroup of breast tumors with elevated expression of tumor-suppressive miRNAs, and we showed that miRNAs can be used as blood-borne biomarkers for detection and staging of breast cancer. The identification of several molecular subtype-specific miRNAs in this study also suggests that blood tests directed at the molecular subtypes can be developed in the future. However, to do so, a larger repository of molecular subtype-specific miRNA expression is required.

## Abbreviations

CTC: circulating tumor cell; FDR: false discovery rate; miRNA: microRNA; PBMC: peripheral blood mononuclear cell; RECIST: response-evaluation criteria in solid tumors; RISC: RNA-induced silencing complex; sRNA: small RNA; SSP: single sample predictor; UHCA: unsupervised hierarchical cluster analysis.

## Competing interests

The authors declare that they have no competing interests.

## Authors' contributions

EVS, MCAW, and IVDA acquired all data. IVDA and SJVL performed data analysis. EVS, MCAW, IVDA, and SVL assisted in data interpretation. IVDA, SJVL, PBV, and LD contributed to the conception and design of the study. EVS, MCAW, IVDA, and SVL were involved in drafting the manuscript. All authors were involved in revising the manuscript critically for important intellectual content. All authors gave final approval of the version to be published.

## Supplementary Material

Additional file 1 **To evaluate assay reproducibility, we tested four samples in duplicate**. The scatterplots demonstrate the result for these samples. The blue line represents the regression line and the correlation coefficients, and corresponding *P *values are given on top of the scatterplot. Click here for file

Additional file 2 **To perform a technical validation of our miRNA-expression data, we analyzed 12 samples by using the nCounter Analysis System and compared these results with the qRT-PCR-based miRNA expression profiles**. The scatterplots illustrate the result of this comparison. The correlation coefficients for each comparison are reported on top of the scatterplots. Click here for file

Additional file 3 **Boxplots showing the comparison of the miRNA-expression profiles of four selected miRNAs (miR-135b, miR-106a, miR-100, and miR-145) between tumor samples grouped by their SSP-defined molecular subtype**. The top row represents two miRNAs overexpressed in the Basal-like samples; the bottom row represents two miRNAs overexpressed in Normal-like samples. The color scheme under each boxplot is adopted from the article by Blenkiron and colleagues and depicts the expression of the corresponding miRNAs according to the SSP-defined molecular subtypes, as reported in their study. Red indicates overexpression, and grey indicates repression. As can be observed, the variation in miRNA expression across the SSP-defined molecular subtypes is in good agreement in both studies. Click here for file

Additional file 4 **Boxplot illustrating the sRNA yields extracted from five different peripheral blood media**. The X-axis depicts the different analyzed media (from left to right: serum, plasma, platelet-rich plasma, peripheral blood mononuclear cells (PBMCs), and whole blood); the Y-axis depicts the sRNA concentration. The sRNA yields are most pronounced in whole blood followed by the PBMC fraction. For serum, plasma, and platelet-rich plasma, the results are comparable, although the sRNA yield is slightly higher in serum. Click here for file

## References

[B1] Ambros V MicroRNA pathways in flies and worms: growth, death, fat, stress, and timing Cell 2003 113 673 676 10.1016/S0092-8674(03)00428-8 12809598

[B2] Bartel DP MicroRNAs: genomics, biogenesis, mechanism, and function Cell 2004 116 281 297 10.1016/S0092-8674(04)00045-5 14744438

[B3] He L Hannon GJ MicroRNAs: small RNAs with a big role in gene regulation Nat Rev Genet 2004 5 522 531 10.1038/nrg1379 15211354

[B4] Lim LP Lau NC Garrett-Engele P Grimson A Schelter JM Castle J Bartel DP Linsley PS Johnson JM Microarray analysis shows that some microRNAs downregulate large numbers of target mRNAs Nature 2005 433 769 773 10.1038/nature03315 15685193

[B5] Sevignani C Calin GA Siracusa LD Croce CM Mammalian microRNAs: a small world for fine-tuning gene expression Mamm Genome 2006 17 189 202 10.1007/s00335-005-0066-3 16518686 PMC2679635

[B6] Zamore PD Haley B Ribo-gnome: the big world of small RNAs Science 2005 309 1519 1524 10.1126/science.1111444 16141061

[B7] Lewis BP Burge CB Bartel DP Conserved seed pairing, often flanked by adenosines, indicates that thousands of human genes are microRNA targets Cell 2005 120 15 20 10.1016/j.cell.2004.12.035 15652477

[B8] Erson AE Petty EM MicroRNAs in development and disease Clin Genet 2008 74 296 306 10.1111/j.1399-0004.2008.01076.x 18713256

[B9] Cheng AM Byrom MW Shelton J Ford LP Antisense inhibition of human miRNAs and indications for an involvement of miRNA in cell growth and apoptosis Nucleic Acids Res 2005 33 1290 1297 10.1093/nar/gki200 15741182 PMC552951

[B10] Xu P Guo M Hay BA MicroRNAs and the regulation of cell death Trends Genet 2004 20 617 624 10.1016/j.tig.2004.09.010 15522457

[B11] Karp X Ambros V Developmental biology: encountering microRNAs in cell fate signaling Science 2005 310 1288 1289 10.1126/science.1121566 16311325

[B12] Pillai RS MicroRNA function: multiple mechanisms for a tiny RNA? RNA 2005 11 1753 1761 10.1261/rna.2248605 16314451 PMC1370863

[B13] Lu J Getz G Miska EA Alvarez-Saavedra E Lamb J Peck D Sweet-Cordero A Ebert BL Mak RH Ferrando AA Downing JR Jacks T Horvitz HR Golub TR MicroRNA expression profiles classify human cancers Nature 2005 435 834 838 10.1038/nature03702 15944708

[B14] Blenkiron C Goldstein LD Thorne NP Spiteri I Chin S-F Dunning MJ Barbosa-Morais NL Teschendorff AE Green AR Ellis IO Tavaré S Caldas C Miska EA MicroRNA expression profiling of human breast cancer identifies new markers of tumor subtype Genome Biol 2007 8 R214 10.1186/gb-2007-8-10-r214 17922911 PMC2246288

[B15] Baffa R Fassan M Volinia S O'Hara B Liu C Palazzo JP Gardiman M Rugge M Gomella LG Croce CM Rosenberg A MicroRNA expression profiling of human metastatic cancers identifies cancer gene targets J Pathol 2009 219 214 221 10.1002/path.2586 19593777

[B16] Volinia S Calin GA Liu CG Ambs S Cimmino A Petrocca F Visone R Iorio M Roldo C Ferracin M Prueitt RL Yanaihara N Lanza G Scarpa A Vecchione A Negrini M Harris CC Croce CM A microRNA expression signature of human solid tumors defines cancer gene targets Proc Natl Acad Sci USA 2006 103 2257 2261 10.1073/pnas.0510565103 16461460 PMC1413718

[B17] Martello G Rosato A Ferrari F Manfrin A Cordenonsi M Dupont S Enzo E Guzzardo V Rondina M Spruce T Parenti AR Daidone MG Bicciato S Piccolo S A microRNA targeting dicer for metastasis control Cell 2010 141 1195 1207 10.1016/j.cell.2010.05.017 20603000

[B18] Ma L Teruya-Feldstein J Weinberg RA Tumour invasion and metastasis initiated by microRNA-10b in breast cancer Nature 2007 449 682 688 10.1038/nature06174 17898713

[B19] Johnson SM Grosshans H Shingara J Byrom M Jarvis R Cheng A Labourier E Reinert KL Brown D Slack FJ RAS is regulated by the let-7 microRNA family Cell 2005 120 635 647 10.1016/j.cell.2005.01.014 15766527

[B20] Fabbri M Garzon R Cimmino A Liu Z Zanesi N Callegari E Liu S Alder H Costinean S Fernandez-Cymering C Volinia S Guler G Morrison CD Chan KK Marcucci G Calin GA Huebner K Croce CM MicroRNA-29 family reverts aberrant methylation in lung cancer by targeting DNA methyltransferases 3A and 3B Proc Natl Acad Sci USA 2007 104 15805 15810 10.1073/pnas.0707628104 17890317 PMC2000384

[B21] Bombonati A Sgroi DC The molecular pathology of breast cancer progression J Pathol 2011 223 307 317 21125683 10.1002/path.2808 PMC3069504

[B22] Perou CM Sørlie T Eisen MB Van De Rijn M Jeffrey SS Rees CA Pollack JR Ross DT Johnsen H Akslen LA Fluge O Pergamenschikov A Williams C Zhu SX Lønning PE Børresen-Dale AL Brown PO Botstein D Molecular portraits of human breast tumours Nature 2000 406 747 752 10.1038/35021093 10963602

[B23] Prat A Parker JS Karginova O Fan C Livasy C Herschkowitz JI He X Perou CM Phenotypic and molecular characterization of the claudin-low intrinsic subtype of breast cancer Breast Cancer Res 2010 12 R68 10.1186/bcr2635 20813035 PMC3096954

[B24] Gatza ML Lucas JE Barry WT Kim JW Wang Q Crawford MD Datto MB Kelley M Mathey-Prevot B Potti A Nevins JR A pathway-based classification of human breast cancer Proc Natl Acad Sci USA 2010 107 6994 6999 10.1073/pnas.0912708107 20335537 PMC2872436

[B25] Iorio MV Ferracin M Liu CG Veronese A Spizzo R Sabbioni S Magri E Pedriali M Fabbri M Campiglio M Ménard S Palazzo JP Rosenberg A Musiani P Volinia S Nenci I Calin GA Querzoli P Negrini M Croce C MicroRNA gene expression deregulation in human breast cancer Cancer Res 2005 65 7065 7070 10.1158/0008-5472.CAN-05-1783 16103053

[B26] Xi Y Nakajima G Gavin E Morris CG Kudo K Hayashi K Ju J Systematic analysis of microRNA expression of RNA extracted from fresh frozen and formalin-fixed paraffin-embedded samples RNA 2007 13 1668 1674 10.1261/rna.642907 17698639 PMC1986820

[B27] Li J Smyth P Flavin R Cahill S Denning K Aherne S Guenther SM O'Leary JJ Sheils O Comparison of miRNA expression patterns using total RNA extracted from matched samples of formalin-fixed paraffin-embedded (FFPE) cells and snap frozen cells BMC Biotechnol 2007 7 1 6 10.1186/1472-6750-7-1 17603869 PMC1914054

[B28] Gilad S Meiri E Yogev Y Benjamin S Lebanony D Yerushalmi N Benjamin H Kushnir M Cholakh H Melamed N Bentwich Z Hod M Goren Y Chajut A Serum microRNAs are promising novel biomarkers PLoS ONE 2008 3 e3148 10.1371/journal.pone.0003148 18773077 PMC2519789

[B29] Mitchell PS Parkin RK Kroh EM Frits BR Wyman SK Pogosova-Agadjanyan EL Peterson A Noteboom J O'Briant KCO Allen A Lin DW Urban N Drescher CW Knudsen BS Stirewalt DL Gentleman R Vessella RL Nelson PS Martin DB Tewari M Circulating microRNAs as stable blood-based markers for cancer detection Proc Natl Acad Sci USA 2008 105 10513 10518 10.1073/pnas.0804549105 18663219 PMC2492472

[B30] Heneghan HM Miller N Lowery AJ Sweeney KJ Newell J Kerin MJ Circulating microRNAs as novel minimally invasive biomarkers for breast cancer Ann Surg 2010 251 499 505 10.1097/SLA.0b013e3181cc939f 20134314

[B31] Van der Auwera I Elst HJ Van Laere SJ Maes H Huget P van Dam P Van Marck EA Vermeulen PB Dirix LY The presence of circulating total DNA and methylated genes is associated with circulating tumour cells in blood from breast cancer patients Br J Cancer 2009 100 1277 1286 10.1038/sj.bjc.6605013 19367284 PMC2676551

[B32] Van der Auwera I Peeters D Benoy IH Elst HJ Van Laere SJ Prové A Maes H Huget P van Dam P Vermeulen PB Dirix LY Circulating tumour cell detection: a direct comparison between the CellSearch System, the AdnaTest and CK-19/mammaglobin RT-PCR in patients with metastatic breast cancer Br J Cancer 2010 102 276 284 10.1038/sj.bjc.6605472 19953098 PMC2816650

[B33] Therasse P Arbuck SG Eisenhauer EA Wanders J Kaplan RS Rubinstein L Verweij J Van Glabbeke M van Oosterom AT Christian MC Gwyther SG New guidelines to evaluate the response to treatment in solid tumors: European Organization for Research and Treatment of Cancer, National Cancer Institute of the United States, National Cancer Institute of Canada J Natl Cancer Inst 2000 92 205 216 10.1093/jnci/92.3.205 10655437

[B34] Sieuwerts AM Mostert B Bolt-de Vries J Peeters D de Jongh FE Stouthard JM Dirix LY van Dam PA Van Galen A de Weerd V Kraan J van der Spoel P Ramírez-Moreno R van Deurzen CH Smid M Yu JX Jiang J Wang Y Gratama JW Sleijfer S Foekens JA Martens JW mRNA and microRNA expression profiles in circulating tumor cells and primary tumors of metastatic breast cancer patients Clin Cancer Res 2011 17 3600 3618 10.1158/1078-0432.CCR-11-0255 21505063

[B35] Mestdagh P Van Vlierberghe P De Weer A Muth D Westermann F Speleman F Vandesompele J A novel and universal method for microRNA RT-qPCR data normalization Genome Biol 2009 10 R64 10.1186/gb-2009-10-6-r64 19531210 PMC2718498

[B36] Livak KJ Schmittgen TD Analysis of relative gene expression data using real-time quantitative PCR and the 2(-Delta Delta C(T)) Method Methods 2001 25 402 408 10.1006/meth.2001.1262 11846609

[B37] Parker JS Mullins M Cheang MC Leung S Voduc D Vickery T Davies S Fauron C He X Hu Z Quackenbush JF Stijleman IJ Palazzo J Marron JS Nobel AB Mardis E Nielsen TO Ellis MJ Perou CM Bernard PS Supervised risk predictor of breast cancer based on intrinsic subtypes J Clin Oncol 2009 27 1160 1167 10.1200/JCO.2008.18.1370 19204204 PMC2667820

[B38] Van Laere S Van der Auwera I Van den Eynden G Van Hummelen P van Dam P Van Marck E Vermeulen PB Dirix L Distinct molecular phenotype of inflammatory breast cancer compared to non-inflammatory breast cancer using Affymetrix-based genome-wide gene-expression analysis Br J Cancer 2007 97 1165 1174 10.1038/sj.bjc.6603967 17848951 PMC2360452

[B39] Van der Auwera I Yu W Suo L Van Neste L van Dam P Van Marck EA Pauwels P Vermeulen PB Dirix LY Van Laere SJ Array-based DNA methylation profiling for breast cancer subtype discrimination PLoS ONE 2010 5 e12616 10.1371/journal.pone.0012616 20830311 PMC2935385

[B40] Van der Auwera I Limame R van Dam P Vermeulen PB Dirix LY Van Laere SJ Integrated miRNA and mRNA expression profiling of the inflammatory breast cancer subtype Br J Cancer 2010 103 532 541 10.1038/sj.bjc.6605787 20664596 PMC2939785

[B41] Cheng C Fu X Alves P Gerstein M mRNA expression profiles show differential regulatory effects of microRNAs between estrogen receptor-positive and estrogen receptor-negative breast cancer Genome Biol 2009 10 R90 10.1186/gb-2009-10-9-r90 19723326 PMC2768979

[B42] Dedes KJ Natrajan R Lambros MB Geyer FC Lopez-Garcia MA Savage K Jones RL Reis-Filho JS Down-regulation of the miRNA master regulators Drosha and Dicer is associated with specific subgroups of breast cancer Eur J Cancer 2011 47 138 150 10.1016/j.ejca.2010.08.007 20832293

[B43] Janssen EAM Slewa A Gudlaugsson E Jonsdottir K Skaland I Søiland H Baak JP Biologic profiling of lymph node negative breast cancers by means of microRNA expression Mod Pathol 2010 23 1567 1576 10.1038/modpathol.2010.177 20818337

[B44] Bockmeyer CL Christgen M Müller M Fischer S Ahrens P Länger F Kreipe H Lehmann U MicroRNA profiles of healthy basal and luminal mammary epithelial cells are distinct and reflected in different breast cancer subtypes Breast Cancer Res Treat 2011 in press 10.1007/s10549-010-1303-3 21409395

[B45] Lowery AJ Miller N Devaney A McNeill RE Davoren PA Lemetre C Benes V Schmidt S Blake J Ball G Kerin MJ MicroRNA signatures predict oestrogen receptor, progesterone receptor and HER2/neu receptor status in breast cancer Breast Cancer Res 2009 11 R27 10.1186/bcr2257 19432961 PMC2716495

[B46] Castellano L Giamas G Jacob J Coombes RC Lucchesi W Thiruchelvam P Barton G Jiao LR Wait R Waxman J Hannon GJ Stebbing J The estrogen receptor-alpha-induced microRNA signature regulates itself and its transcriptional response Proc Natl Acad Sci USA 2009 106 15732 15737 10.1073/pnas.0906947106 19706389 PMC2747188

[B47] Liu S Goldstein RH Scepansky EM Rosenblatt M Inhibition of rho-associated kinase signaling prevents breast cancer metastasis to human bone Cancer Res 2009 69 8742 8751 10.1158/0008-5472.CAN-09-1541 19887617

[B48] Li H Bian C Liao L Li J Zhao RC miR-17-5p promotes human breast cancer cell migration and invasion through suppression of HBP1 Breast Cancer Res Treat 2011 126 565 575 10.1007/s10549-010-0954-4 20505989

[B49] Ma X Becker Buscaglia LE Barker JR Li Y MicroRNAs in NF-kappaB signaling J Mol Cell Biol 2011 3 159 166 10.1093/jmcb/mjr007 21502305 PMC3104013

[B50] Herschkowitz JI Zhao W Zhang M Usary J Murrow G Edwards D Knezevic J Greene SB Darr D Troester MA Hilsenbeck SG Medina D Perou CM Rosen JM Breast Cancer Special Feature: Comparative oncogenomics identifies breast tumors enriched in functional tumor-initiating cells Proc Natl Acad Sci USA 2011 in press 10.1073/pnas.1018862108 PMC3286979 21633010

[B51] Gregory PA Bracken CP Smith E Bert AG Wright JA Roslan S Morris M Wyatt L Farshid G Lim YY Lindeman GJ Shannon MF Drew PA Khew-Goodall Y Goodall GJ An autocrine TGF-beta/ZEB/miR-200 signaling network regulates establishment and maintenance of epithelial-mesenchymal transition Mol Biol Cell 2011 22 1686 1698 10.1091/mbc.E11-02-0103 21411626 PMC3093321

[B52] Gregory PA Bert AG Paterson EL Barry SC Tsykin A Farshid G Vadas MA Khew-Goodall Y Goodall GJ The miR-200 family and miR-205 regulate epithelial to mesenchymal transition by targeting ZEB1 and SIP1 Nature 2008 10 593 601 10.1038/ncb1722 18376396

[B53] Yu F Yao H Zhu P Zhang X Pan Q Gong C Huang Y Hu X Su F Lieberman J Song E let-7 regulates self renewal and tumorigenicity of breast cancer cells Cell 2007 131 1109 1123 10.1016/j.cell.2007.10.054 18083101

[B54] Song B Wang Y Titmus MA Botchkina G Formentini A Kornmann M Ju J Molecular mechanism of chemoresistance by miR-215 in osteosarcoma and colon cancer cells Mol Cancer 2010 9 96 10.1186/1476-4598-9-96 20433742 PMC2881118

[B55] Duan Z Person RE Lee H-H Huang S Donadieu J Badolato R Grimes HL Papayannopoulou T Horwitz MS Epigenetic regulation of protein-coding and microRNA genes by the Gfi1-interacting tumor suppressor PRDM5 Mol Cell Biol 2007 27 6889 6902 10.1128/MCB.00762-07 17636019 PMC2099216

[B56] Fang X Yoon J-G Li L Yu W Shao J Hua D Zheng S Hood L Goodlett DR Foltz G Lin B The SOX2 response program in glioblastoma multiforme: an integrated ChIP-seq, expression microarray, and microRNA analysis BMC Genomics 2011 12 11 10.1186/1471-2164-12-11 21211035 PMC3022822

[B57] Lavon I Zrihan D Granit A Einstein O Fainstein N Cohen MA Cohen MA Zelikovitch B Shoshan Y Spektor S Reubinoff BE Felig Y Gerlitz O Ben-Hur T Smith Y Siegal T Gliomas display a microRNA expression profile reminiscent of neural precursor cells Neuro Oncol 2010 12 422 433 20406893 10.1093/neuonc/nop061 PMC2940621

[B58] Hunter MP Ismail N Zhang X Aguda BD Lee EJ Yu L Xiao T Schafer J Lee ML Schmittgen TD Nana-Sinkam SP Jarjoura D Marsh CB Detection of microRNA expression in human peripheral blood microvesicles PLoS ONE 2008 3 e3694 10.1371/journal.pone.0003694 19002258 PMC2577891

[B59] Arroyo JD Chevillet JR Kroh EM Ruf IK Pritchard CC Gibson DF Mitchell PS Bennett CF Pogosova-Agadjanyan EL Stirewalt DL Tait JF Tewari M Argonaute2 complexes carry a population of circulating microRNAs independent of vesicles in human plasma Proc Natl Acad Sci USA 2011 108 5003 5008 10.1073/pnas.1019055108 21383194 PMC3064324

[B60] Vickers KC Palmisano BT Shoucri BM Shamburek RD Remaley AT MicroRNAs are transported in plasma and delivered to recipient cells by high-density lipoproteins Nat Cell Biol 2011 13 423 433 10.1038/ncb2210 21423178 PMC3074610

[B61] Valadi H Ekström K Bossios A Sjöstrand M Lee JJ Lötvall JO Exosome-mediated transfer of mRNAs and microRNAs is a novel mechanism of genetic exchange between cells Nat Cell Biol 2007 9 654 659 10.1038/ncb1596 17486113

[B62] Muralidharan-Chari V Clancy JW Sedgwick A D'Souza-Schorey C Microvesicles: mediators of extracellular communication during cancer progression J Cell Sci 2010 123 1603 1611 10.1242/jcs.064386 20445011 PMC2864708

[B63] Pigati L Yaddanapudi SC Iyengar R Kim DJ Hearn SA Danforth D Hastings ML Duelli DM Selective release of microRNA species from normal and malignant mammary epithelial cells PLoS ONE 2010 5 e13515 10.1371/journal.pone.0013515 20976003 PMC2958125

