# Supplementary material for: Expression profiling of cancerous and normal breast tissues identifies microRNAs that are differentially expressed in serum from patients with (metastatic) breast cancer and healthy volunteers
Source: Breast Cancer Res. 2012 Feb 21;14(1):R34. doi: 10.1186/bcr3127 (PMC3496152; doi:10.1186/bcr3127)

Additional file 1

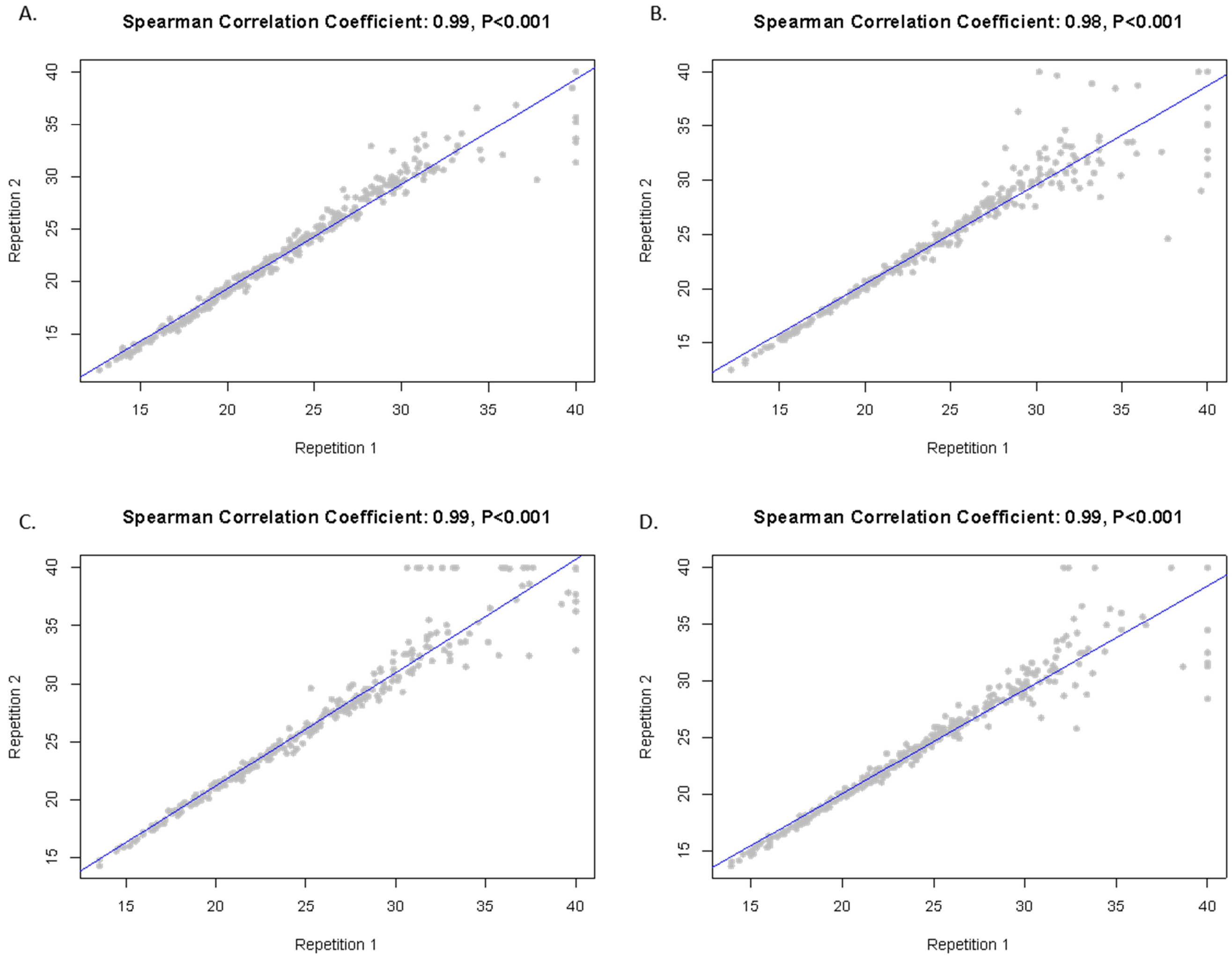

Supplement: Additional file 1 — To evaluate assay reproducibility, we tested four samples in duplicate. The scatterplots demonstrate the result for these samples. The blue line represents the regression line and the correlation coefficients, and corresponding P values are given on top of the scatterplot. [file bcr3127-S1.PDF]
