# Supplementary figures and images for: Expression profiling of cancerous and normal breast tissues identifies microRNAs that are differentially expressed in serum from patients with (metastatic) breast cancer and healthy volunteers
Source: Breast Cancer Res. 2012 Feb 21;14(1):R34. doi: 10.1186/bcr3127 (PMC3496152; doi:10.1186/bcr3127)

## Additional file 2

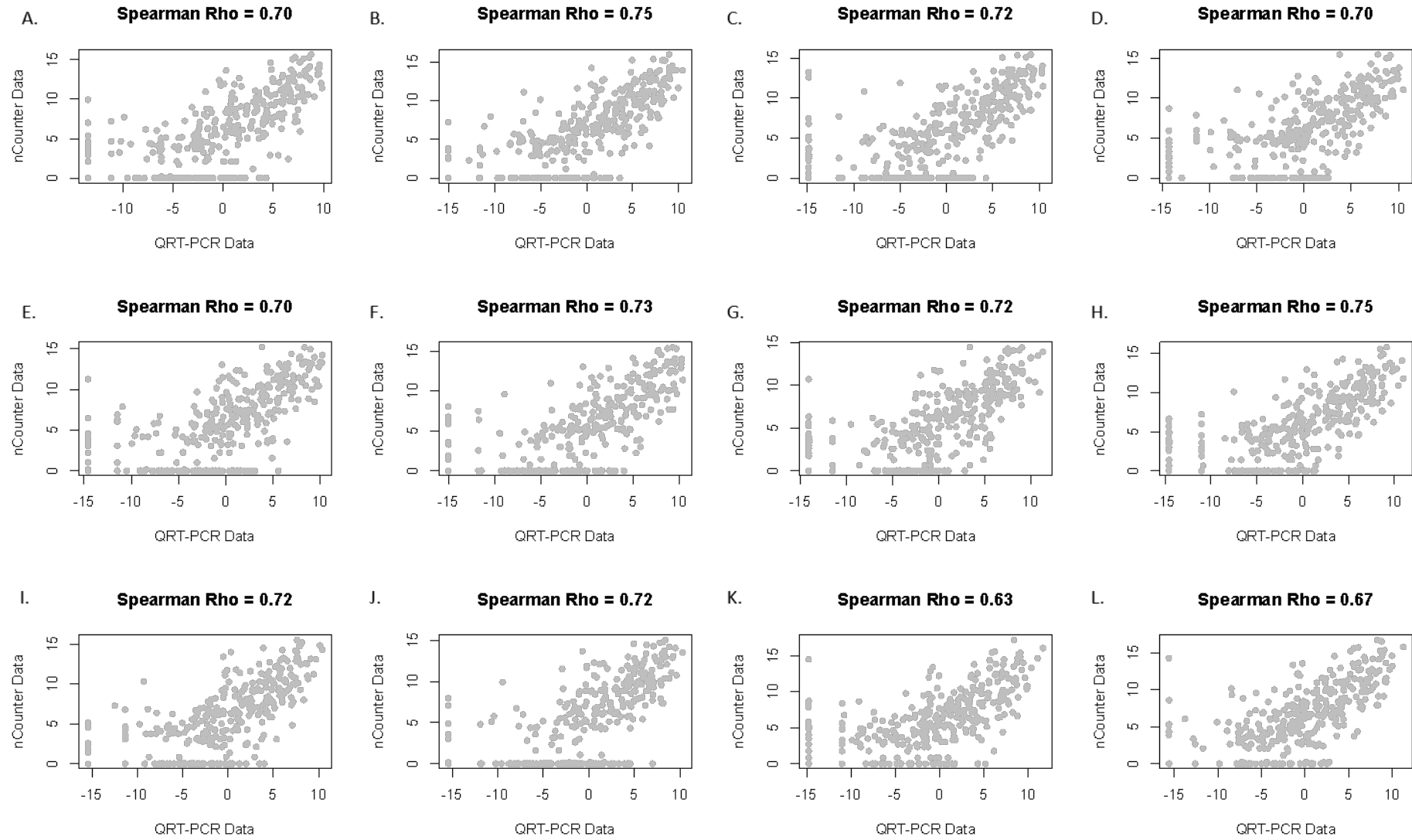

Supplement: Additional file 2 — To perform a technical validation of our miRNA-expression data, we analyzed 12 samples by using the nCounter Analysis System and compared these results with the qRT-PCR-based miRNA expression profiles. The scatterplots illustrate the result of this comparison. The correlation coefficients for each comparison are reported on top of the scatterplots. [file bcr3127-S2.PDF]

Additional file 3

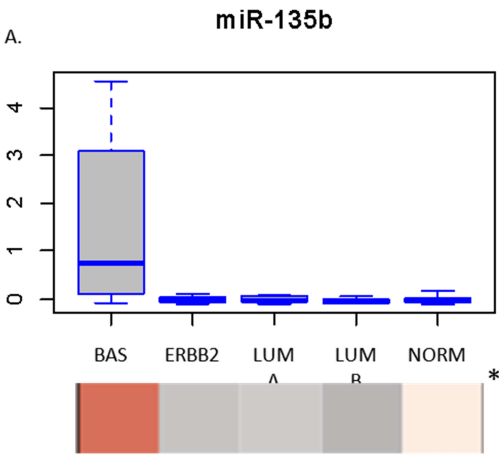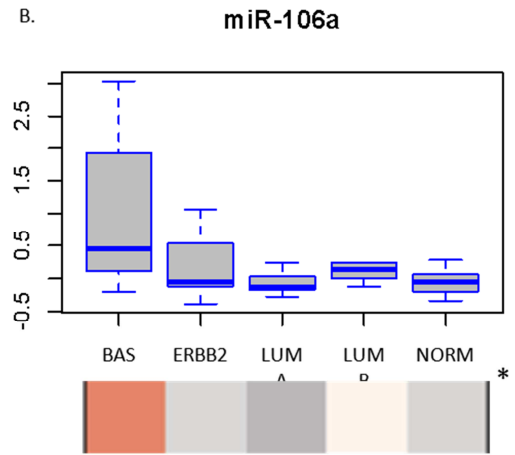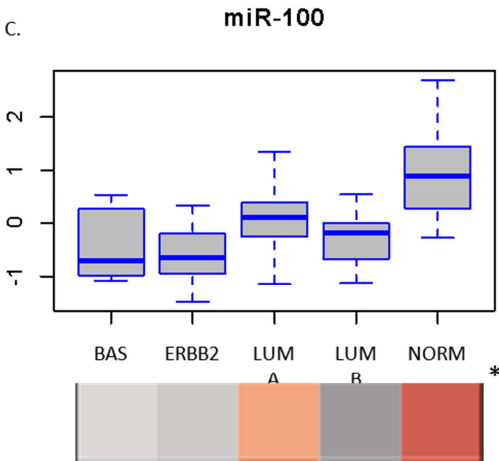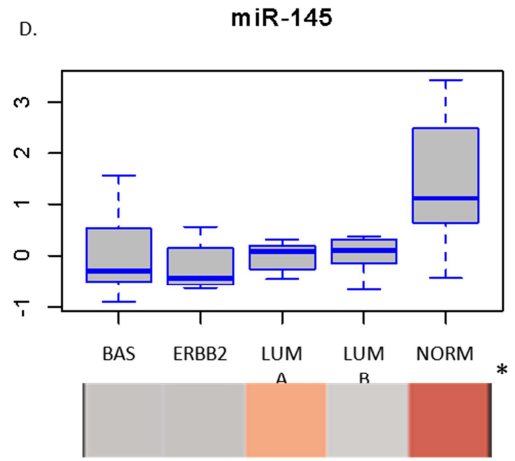

Supplement: Additional file 3 — Boxplots showing the comparison of the miRNA-expression profiles of four selected miRNAs (miR-135b, miR-106a, miR-100, and miR-145) between tumor samples grouped by their SSP-defined molecular subtype. The top row represents two miRNAs overexpressed in the Basal-like samples; the bottom row represents two miRNAs overexpressed in Normal-like samples. The color scheme under each boxplot is adopted from the article by Blenkiron and colleagues and depicts the expression of the corresponding miRNAs according to the SSP-defined molecular subtypes, as reported in their study. Red indicates overexpression, and grey indicates repression. As can be observed, the variation in miRNA expression across the SSP-defined molecular subtypes is in good agreement in both studies. [file bcr3127-S3.PDF]

#### Additional file 4

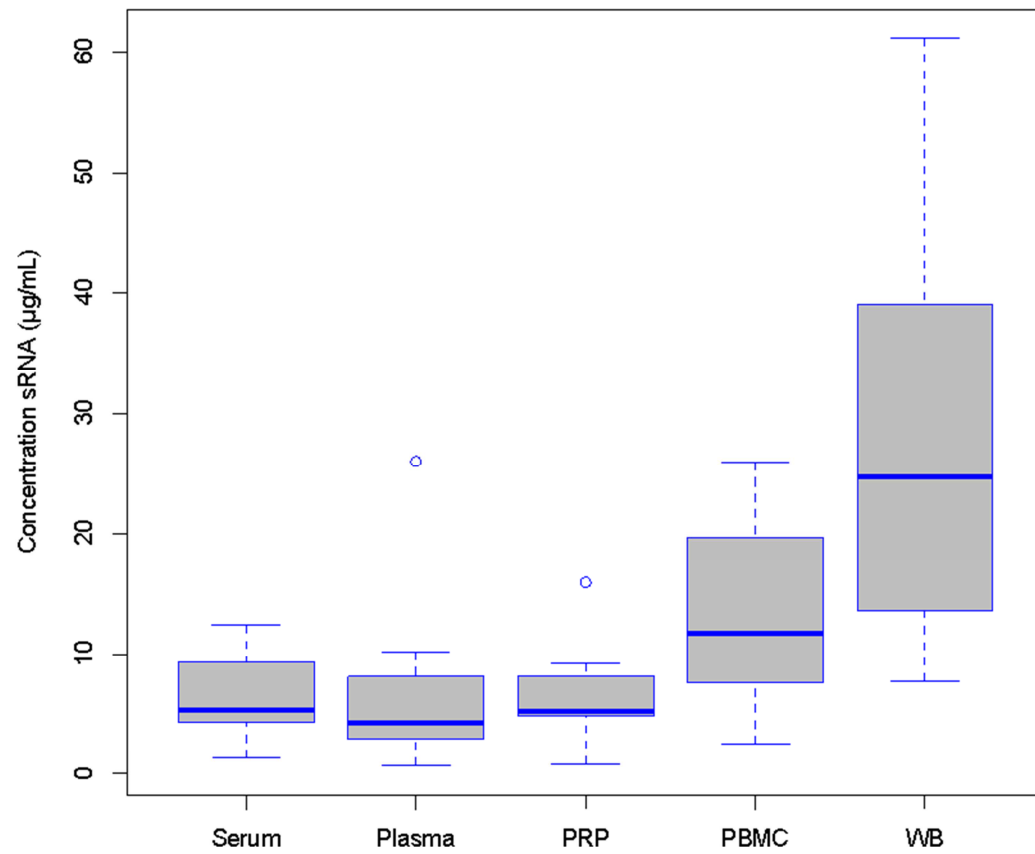

Supplement: Additional file 4 — Boxplot illustrating the sRNA yields extracted from five different peripheral blood media. The X-axis depicts the different analyzed media (from left to right: serum, plasma, platelet-rich plasma, peripheral blood mononuclear cells (PBMCs), and whole blood); the Y-axis depicts the sRNA concentration. The sRNA yields are most pronounced in whole blood followed by the PBMC fraction. For serum, plasma, and platelet-rich plasma, the results are comparable, although the sRNA yield is slightly higher in serum. [file bcr3127-S4.PDF]
